# Expression of the Metastasis Suppressor KAI1 in Uveal Melanoma

**DOI:** 10.1155/2013/683963

**Published:** 2013-08-01

**Authors:** Shawn C. Maloney, Bruno F. Fernandes, Rafaella Cleto Penteado, Emilia Antecka, Vasco Bravo-Filho, Debra Meghan Sanft, Miguel N. Burnier

**Affiliations:** The Henry C. Witelson Ocular Pathology Laboratory, McGill University, 3775 University Street, Room 216, Montreal, QC, Canada H3A 2B4

## Abstract

*Introduction*. Uveal melanoma (UM) is an intraocular tumor that leads to metastatic disease in approximately 50% of afflicted patients. There is no efficacious treatment for metastatic disease in this cancer. Identification of markers that can offer prognostic and therapeutic value is a major focus in this field at present. KAI1 is a metastasis suppressor gene that has been reported to play a role in various human malignancies, although it has not previously been evaluated in UM. *Purpose*. To investigate the expression of KAI1 in UM and its potential value as a prognostic marker. *Materials and Methods*. 18 cases of human primary UM were collected and immunostained for KAI1 expression. A pathologist evaluated staining intensity and distribution semiquantitatively. Each case was categorized as group 1 (low staining) or group 2 (high staining). *Results*. In group 2, two of the 12 cases presented with metastasis. Conversely, in group 1, five out of 6 cases had metastasis. The mean follow-up of patients who did not develop metastasis was 81.81 months (median: 75 months) versus 42.14 months (median: 44 months) for patients with metastasis. *Conclusions*. KAI1 is a promising candidate marker that may offer prognostic value in UM; it may also represent a therapeutic target in metastatic disease.

## 1. Introduction

Uveal melanoma (UM) is the most common primary intraocular malignant tumor in adults, as well as the most common noncutaneous melanoma. This tumor primarily affects Caucasians and has an incidence of approximately 6 cases per million [1]. Metastasis—the leading cause of death in UM patients—can be present at the same time as the diagnosis of the primary tumor or several years thereafter [2, 3]. Tumor metastasis occurs via hematogenous dissemination, with the liver being the most common organ affected, followed by lung and bone [4, 5].

Metastasis suppressor genes (MSGs) are known to play a role in numerous cancers. They are of particular interest to researchers given that they can be implicated in various steps of the metastatic cascade and thus may have therapeutic value. Expression of MSGs is frequently reduced in highly metastatic tumor cells [6]. The MSG KAI1 has previously been shown to interfere with multiple steps of the metastatic cascade, including proliferation, invasion, and migration, making it an attractive marker to evaluate in UM and other cancers [7, 8]. Multiple studies have demonstrated that the expression of KAI1 in some primary tumor types is inversely correlated with formation of metastasis [9]. The aim of this study was to investigate the expression of KAI1 in cases of primary human uveal melanomas and to determine possible correlations with the development of metastatic disease.

## 2. Materials and Methods

Formalin-fixed, paraffin-embedded sections from 18 enucleated eyes of UM patients were used in this study. Inclusion criteria included the following: (1) diagnosis of choroidal melanoma, (2) minimum follow-up of 12 months, and (3) sufficient material for immunohistochemical analysis.

Immunohistochemistry was completed using the Ventana BenchMark fully automated machine. KAI1 staining was performed according to the manufacturer's instructions. The processing of barcode-labeled slides included baking of the slides, solvent-free deparaffinization, and CC1 (Tris-EDTA buffer, pH 8.0) antigen retrieval. Slides were incubated with a KAI1 antibody (Santa Cruz Biotechnology, sc-17752, G-2, 1 : 100) for 30 minutes at 37°C, followed by application of a biotinylated secondary antibody (8 minutes at 37°C) and an avidin-alkaline phosphatase enzyme conjugate complex (8 minutes at 37°C). Finally, the antibody was detected by Fast Red chromogenic substrate and counterstained with hematoxylin. As positive controls, sections of breast cancer were used. For negative controls, the primary antibody was omitted. Sections of choroidal melanomas were graded semiquantitatively for KAI1 expression based on the intensity (negative = 0; mild = 1; strong = 2) and extent (negative = 0; focal, <30%  of  cells positive = 1; diffuse, >30%  of  cells positive = 2) of staining. A combined score was used to separate the patients into two groups according to staining characteristics: group 1, total score of 0 to 2, and group 2, total score of 3 or 4.

All data was in accordance with the Declaration of Helsinki.

## 3. Results

Eighteen cases of uveal melanoma were studied and the patients were followed up after enucleation for an average of 66.38 months (median: 66 months) (Table 1). The patients were divided into two groups according to the staining score. From the six cases in group 1, three did not express KAI1. The mean follow-up time in this group was 56.83 months (median: 54 months), compared to 71.16 months (median: 72.5 months) in group 2.

**Figure 1:**
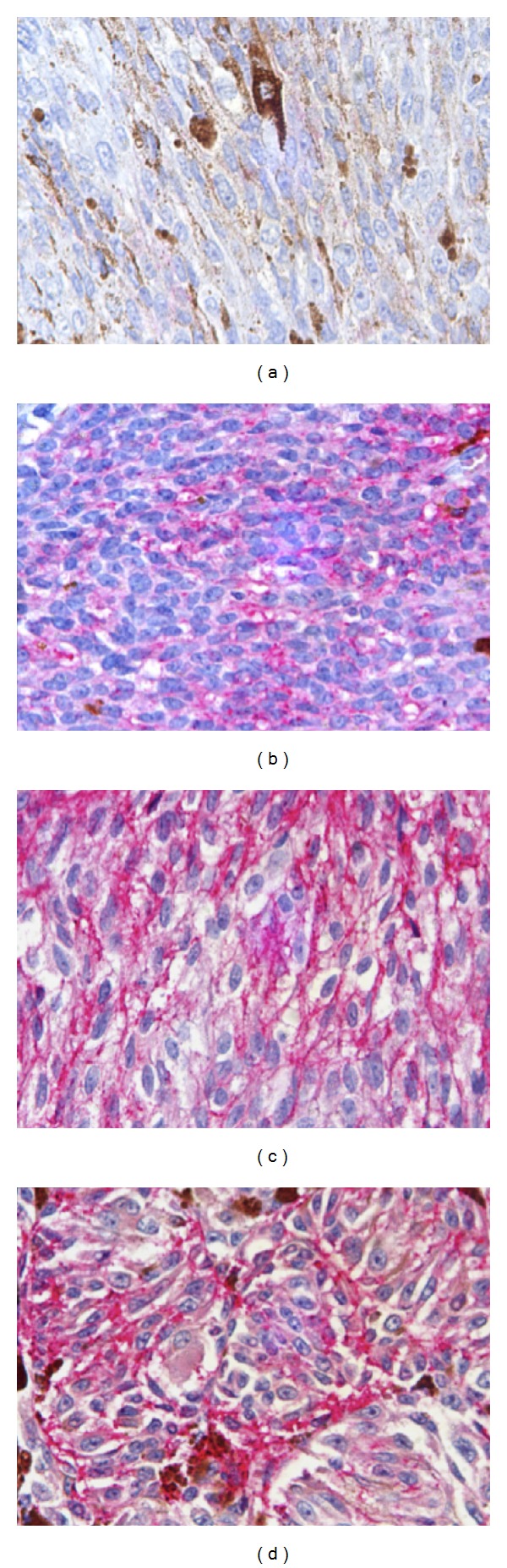
KAI1 in uveal melanoma. (a) Mild and focal cytoplasmic positivity in spindle cell malignant melanoma (640x). (b) Mild and diffuse cytoplasmic positivity in epithelioid malignant melanoma (640x). (c) Strong and diffuse cytoplasmic positivity in mixed (spindle-epithelioid) malignant melanoma (640x). (d) Strong and diffuse cytoplasmic positivity in epithelioid malignant melanoma (640x).

In the high staining group, 2 of the 12 cases presented with metastasis. Conversely, in the low staining group, 5 out of 6 cases had metastasis. The mean follow-up of patients who did not develop metastasis was 81.81 months (median: 75 months) versus 42.14 months (median: 44 months) for patients with metastasis (Figure 1).

To determine whether or not there was a statistically significant association between low KAI1 staining and the development of metastasis, we used Fisher's exact test. We found a significant association (*P* value of 0.0072); however, since the sample size was small, the statistical power of this test is low (0.179).

## 4. Discussion

It is believed that downregulation of MSGs is a critical step to enable tumor cells to complete the metastatic cascade. To date, a total of thirteen MSGs have been characterized in cancer in general, although research on the potential prognostic and therapeutic value of MSGs in UM is not well characterized [10, 11].

To the best of our knowledge, this is the first time that KAI1 has been characterized in uveal melanoma. Although it is known that metastasis can occur several years after the diagnosis of the primary tumor [2], mean follow-up time of patients in this study that did not present with metastasis was nearly twice as long as the follow-up time of patients with metastatic disease. This difference in follow-up time is related to the relatively short survival time for patients diagnosed with metastasis.

In this study, patients that had a combined score of 3 or 4 were significantly less likely to develop metastasis. These data corroborate previous studies that similarly demonstrated such association in other malignancies such as prostate [12], gastric [13], colon [14], cervical [15], breast [16], skin [17], bladder [17], lung [18], pancreatic [19], liver [20], and thyroid cancers [21].

While much has been learned about KAI1, a lot remains to be determined about its function, particularly with respect to its role as a metastasis suppressor. Nevertheless, the initial findings presented in this study highlight KAI1 as a putative regulator of metastasis in UM patients. More research is needed to determine the potential of KAI1 as a diagnostic marker or clinical target in UM.

## 5. Conclusion

A limitation of the current study is the paucity of specimens available for evaluation. This is due to changes in the clinical management of UMs, many of which are treated with brachytherapy rather than undergoing enucleation. Thus, while these preliminary results point to an association between low staining of KAI1 and the development of metastasis, more cases will need to be studied to achieve a higher power result. Given that fine-needle aspiration biopsy (FNAB) of UM is becoming more commonplace, the identification of putative prognostic markers that can be evaluated in FNAB specimens has important clinical implications. Further research will confirm whether or not KAI1 could serve as such a prognostic marker in UM.

## Figures and Tables

**Table 1:** KAI1 expression in uveal melanoma.

	Extent	Intensity	Total score	Metastasis	Follow-up time
Group 1 (6 patients)					
Patient 1	Negative	Negative	0	Liver	44
Patient 2	Focal	Mild	2	Liver	83
Patient 3	Negative	Negative	0	Liver and bone	60
Patient 4	Negative	Negative	0	Liver	48
Patient 5	Focal	Mild	2	No	82
Patient 6	Focal	Mild	2	Liver	24
Group 2 (12 patients)					
Patient 7	Diffuse	Strong	4	No	177
Patient 8	Diffuse	Mild	3	No	72
Patient 9	Focal	Strong	3	No	27
Patient 10	Diffuse	Mild	3	Liver	24
Patient 11	Focal	Strong	3	No	140
Patient 12	Diffuse	Strong	4	No	96
Patient 13	Diffuse	Strong	4	No	86
Patient 14	Diffuse	Strong	4	No	60
Patient 15	Diffuse	Strong	4	No	73
Patient 16	Diffuse	Mild	3	Liver	12
Patient 17	Diffuse	Strong	4	No	75
Patient 18	Focal	Strong	3	No	12
